# Endoscopic findings in the upper gastrointestinal tract in patients with Crohn’s disease are common, highly specific, and associated with chronic gastritis

**DOI:** 10.1038/s41598-022-21630-5

**Published:** 2023-01-13

**Authors:** Katarzyna Graca-Pakulska, Wojciech Błogowski, Iwona Zawada, Anna Deskur, Krzysztof Dąbkowski, Elżbieta Urasińska, Teresa Starzyńska

**Affiliations:** 1 Department of Gastroenterology, Pomeranian Medical University, Szczecin, Poland; 2 Institute of Medical Sciences, University of Zielona Góra, ul. Zyty 28, 65-046 Zielona Gora, Poland; 3 Department of Pathology, Pomeranian Medical University, Szczecin, Poland

**Keywords:** Crohn's disease, Oesophagogastroscopy

## Abstract

Crohn’s disease (CD) may affect the entire gastrointestinal tract including its upper part. However, this aspect is poorly addressed in scientific literature and considered a rare finding. Here we aimed to prospectively investigate the prevalence, characteristics and clinical significance of upper gastrointestinal tract involvement in patients with CD, with particular focus on stomach bamboo joint-like appearance (BJA), *Helicobacter* *pylori* status and presence of microscopic changes. 375 prospectively recruited patients were included. In CD patients the prevalence of gastric and duodenal, but not esophageal, mucosal lesions, such as gastric mucosal inflammation, duodenal edema, ulcerations, and duodenal bulb deformation was significantly higher (at least *p* < 0.01 for all). Similar results were found when only *H.* *pylori* negative individuals were analyzed. Moreover, BJA of the stomach and in case of *H.* *pylori* negative patients also duodenal bulb deformation were detected exclusively in CD patients. Presence of BJA lesion was not significantly associated with neither duration of the disease nor use/history of biologic treatment. Despite absence of *H.* *pylori* infection microscopic features of chronic gastritis were found in almost all (93.5%) patients, and in 31% of controls (*p* < 0.00001). Our analysis outlines that upper gastrointestinal tract involvement in CD is a very common event and frequently manifests with a highly specific BJA lesion. Furthermore, our study reveals that in almost all CD patients features of *H.* *pylori* negative gastritis are present.

## Introduction

Crohn’s disease constitutes an important clinical problem and challenge. It is a chronic and relapsing inflammatory condition of the gastrointestinal (GI) tract that may lead to life-threatening complications. An increasing tendency in prevalence of this disease is constantly observed among predominantly younger individuals in Europe and the USA, where it is estimated that approximately 1.6–3 million patients are affected by this disease. To date, multiple factors are known to be associated with Crohn’s disease, and these include alterations in gut microbiome, abnormal immune response and genetic predisposition (reviewed in detail in^1^). However, despite much research over the last few decades the exact aetiology of the Crohn’s disease remains to be defined, and definitive treatment is still not established.

Crohn’s disease most commonly causes inflammation of the terminal ileum and colon; the current Montreal classification accurately characterizes the affected sites as ileal (L1), colonic (L2), and/or ileocolonic (L3)^1,2^. However, this condition may affect other, more proximal segments of the GI tract, including the upper GI tract, which are accessible and can be visualized during gastroduodenoscopy (esophagus, stomach, duodenum), as well as more distal sites, such as the jejunum or proximal ileal segments. Currently, this clinical presentation is defined by the Montreal classification as L4 involvement.

Until the end of the 90 s, lesions involving the upper GI tract (esophagus, stomach, and/or duodenum) were considered a rare manifestation of Crohn’s disease that were reported to occur in only 4–10% of patients^3,4^. However, Yokota et al.^5^ were the first to observe that upper GI tract involvement may be common in patients with Crohn’s disease (reaching up to 54% of patients), and recent studies^6,7^ have reported similar findings. These preliminary reports highlight that “bamboo joint-like appearance” (BJA) of the gastric folds serves as a potential pathognomonic endoscopic biomarker of this inflammatory bowel disease (IBD)^5–7^. Nonetheless, the prevalence of upper GI tract involvement in Crohn’s disease has been reported based on retrospective analyses, and these offered data with large (12–80%) variations. Moreover, the clinical significance of the BJA phenomenon and mechanisms behind this condition remain to be defined.

In this study we prospectively investigated the prevalence, as well as the characteristic and clinical importance of upper gastrointestinal tract involvement in patients with Crohn’s disease, with particular focus on BJA lesions, *Helicobacter* *pylori* status and presence of microscopic changes in stomach. The results were compared with non-IBD individuals. We hypothesized that the prevalence of gastric and duodenal lesions is significantly higher in patients with Crohn’s disease than it is commonly perceived. Additionally, we suspected that BJA lesions are exclusively observed in patients with Crohn’s disease, and these may be present upon initial diagnosis and/or within the early years of the disease. Finally, we hypothesized that despite lack of *H. pylori* infection patients with Crohn’s disease have microscopic features of gastritis.

## Results

### Prevalence of H. pylori infection among prospectively recruited individuals

**Figure 1:**
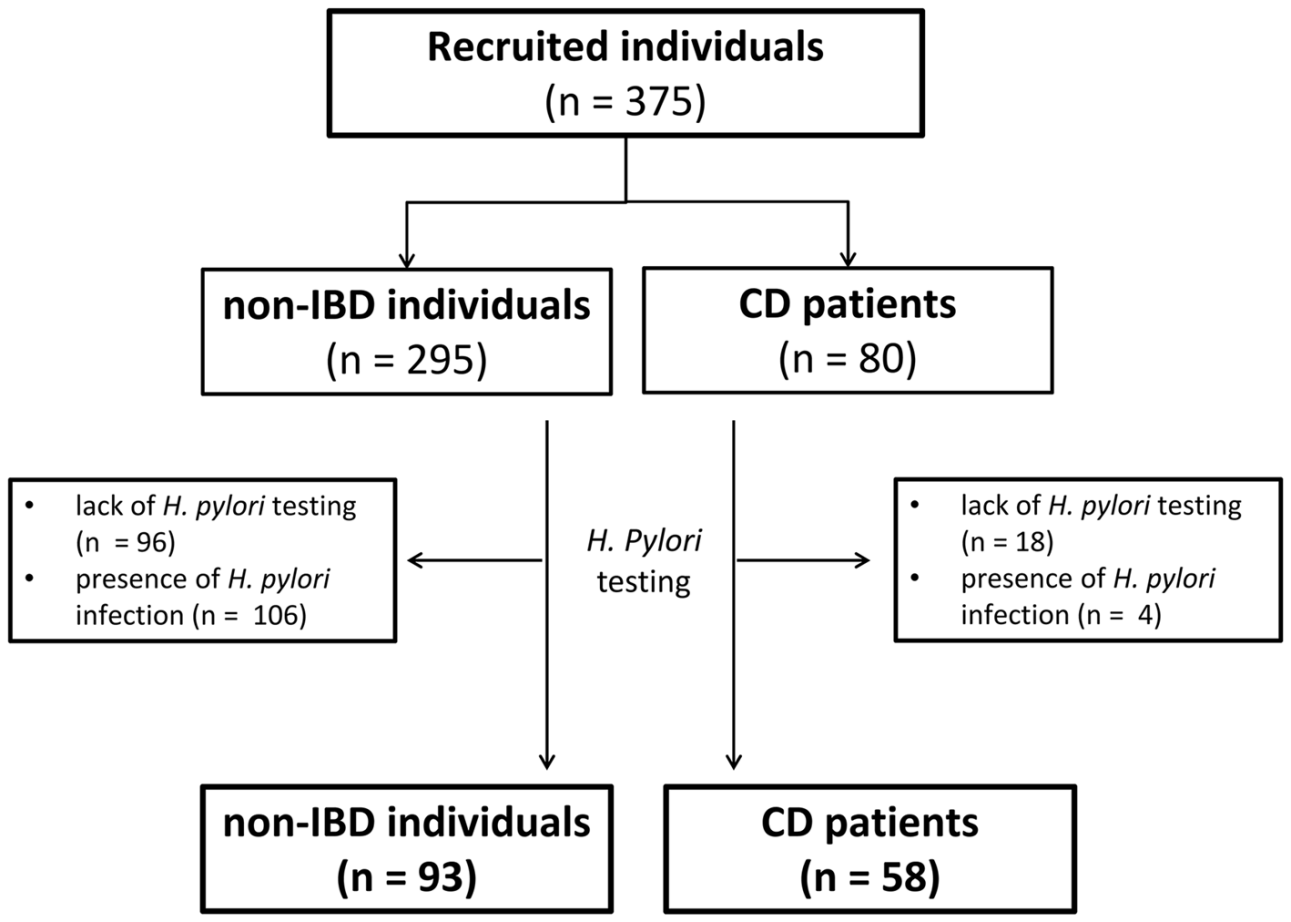
Chart demonstrating flow of patients initially recruited to the study and those included into the final analyses after evaluation of the status of *H. pylori* infection. *CD* Crohn’s disease, *IBD* inflammatory bowel disease.

As demonstrated on Fig. 1 we observed significantly lower prevalence of *H. pylori* infection among the initially recruited patients with Crohn’s disease than in non-IBD generally healthy individuals (6.5% vs. 53.3%, *p* < 0.0000001). As *H. pylori* incidence may increase with age we compared the age of those who were *H. pylori* positive with those who tested negative for infection in both control and IBD individuals. We found no statistically significant differences in this context for either control (*p* = 0.31) or study participants (*p* = 0.89).

### Esophageal lesions

In our study no significant intergroup differences were observed in the prevalence of esophageal erosions, ulcerations, or Barrett’s metaplasia (Supplementary Table 1). However, our analyses also demonstrated that the prevalence of reflux esophagitis was significantly higher in non-IBD individuals (Table 1).

**Table 1:** Significant differences in the prevalence of certain types of endoscopic findings among patients with Crohn’s disease and healthy individuals (together with statistical comparison).

	All			*H. pylori* negative		
	non-IBD individuals (n = 295)	CD patients (n = 80)	*p*	non-IBD individuals (n = 93)	CD patients (n = 58)	*p*
*Esophagus*						
Reflux esophagitis	57 (17.6%)	10 (12.5%)	0.0002	19 (20.4%)	1 (1.7%)	0.0004
*Stomach*						
Mucosal erosions	27 (9.2%)	16 (20%)	0.008	12 (12.9%)	4 (6.9%)	0.05
Mucosal macroscopic inflammation	70 (23.7%)	42 (52.5%)	0.000001	32 (34.4%)	39 (67.2%)	0.00008
BJA	0 (0.00%)	37 (46.3%)	0.0000001	0 (0.0%)	32 (55.2%)	0.0000001
*Duodenum*						
Aphthous lesions	3 (1%)	4 (5%)	0.05	0 (0.0%)	3 (5.2%)	0.05
Bulb deformation	11 (3.7%)	5 (6.3%)	0.24	0 (0.0%)	4 (6.9%)	0.02
Ulcerations	20 (6.8%)	14 (17.5%)	0.005	2 (2.2%)	12 (20.7%)	0.0002
Mucosal swelling	9 (3.1%)	10 (12.5%)	0.002	2 (2.2%)	8 (13.8%)	0.007

Analyses were conducted separately for whole population of recruited individuals and separately for *H. pylori* negative population of non-IBD and CD patients. *BJA* bamboo joint-like appearance, *CD* Crohn’s disease, *IBD* inflammatory bowel disease.

*p* level of significance.

### Gastric lesions

**Figure 2:**
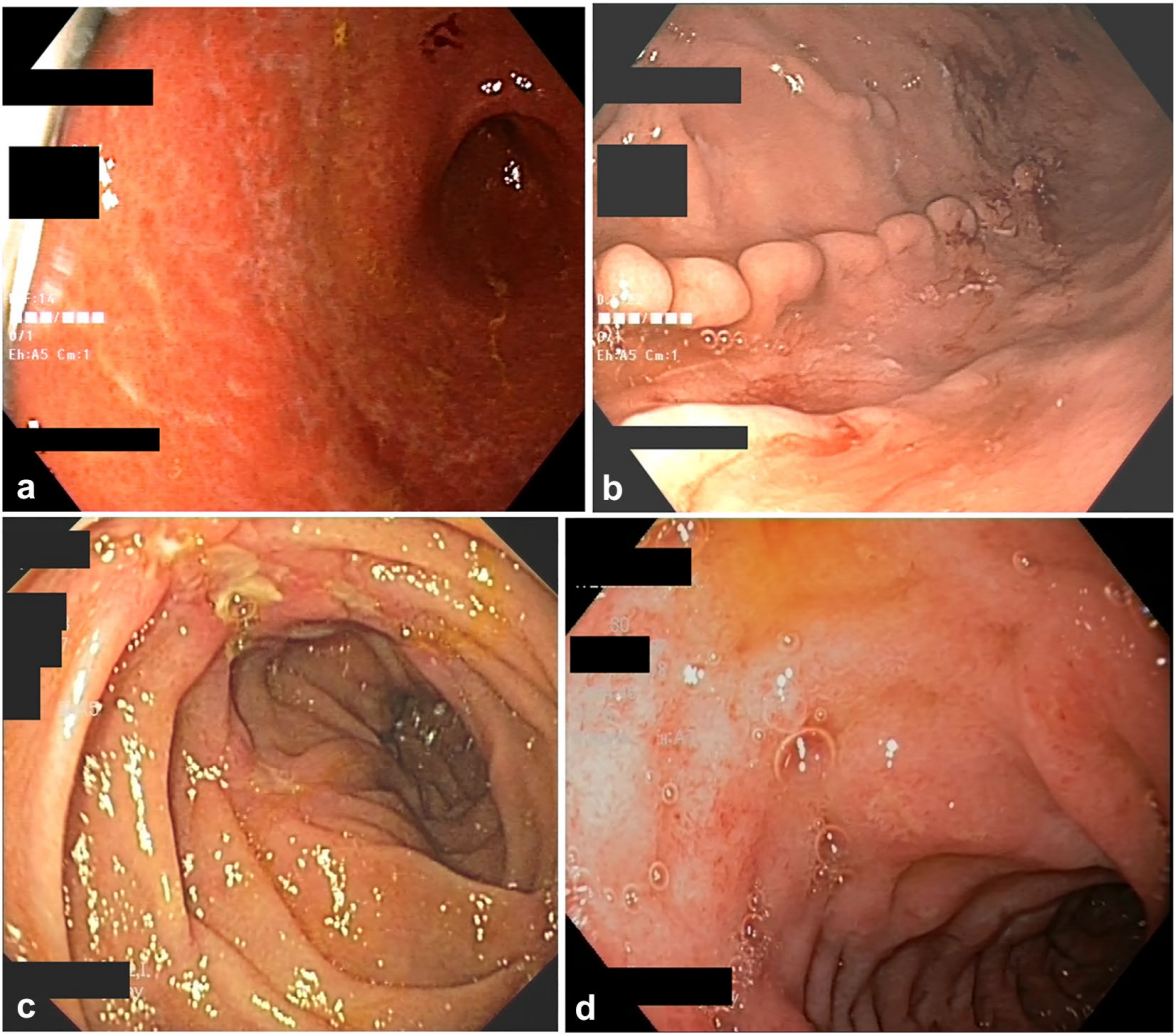
Representative images of certain significant endoscopic findings within the upper gastrointestinal tract in patients with Crohn’s disease. (**a**) gastric mucosal inflammation, (**b**) bamboo joint-like appearance of the stomach, (**c**) ulcerations in descending part of duodenum, (**d**) duodenal mucosal inflammation and erosions.

**Figure 3:**
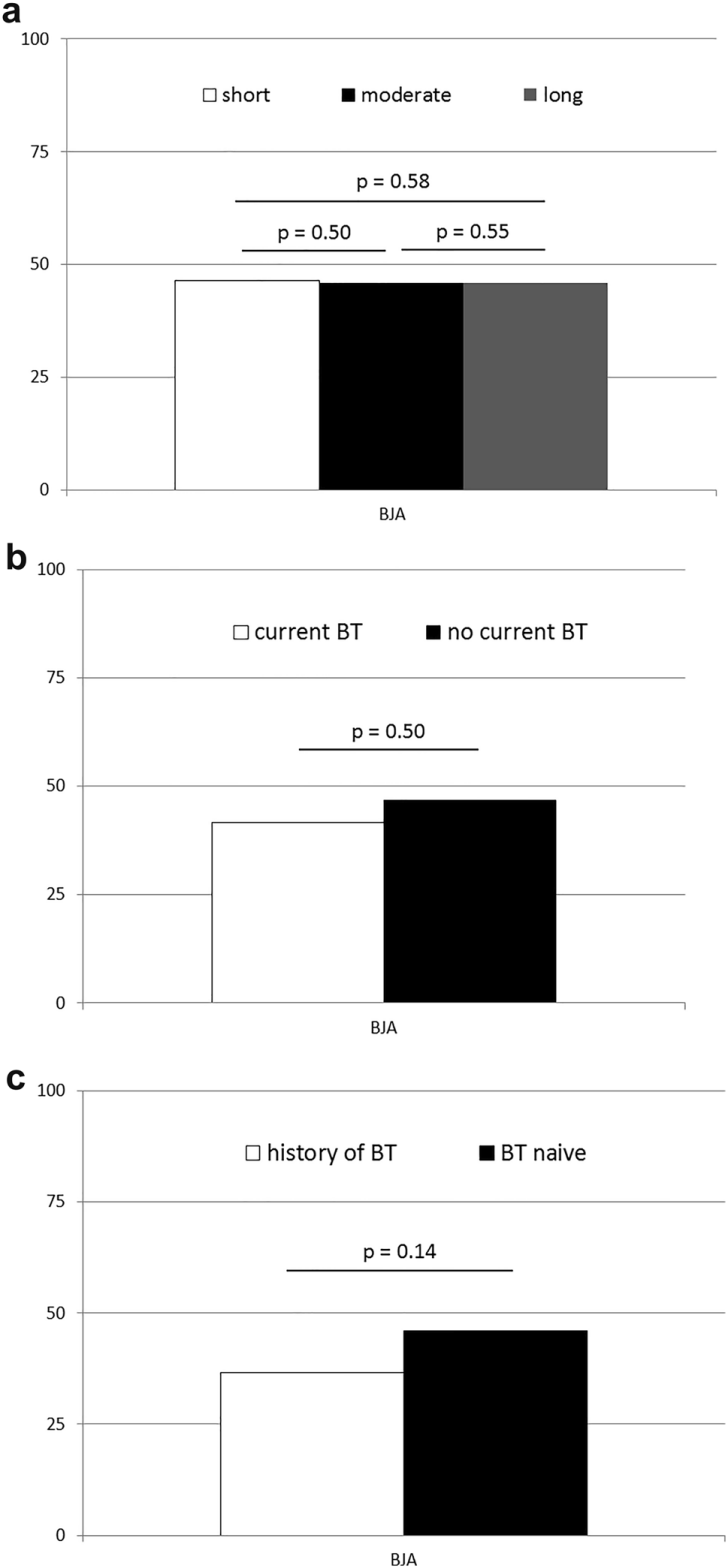
Prevalence of bamboo joint-like appearance (BJA) of the stomach in subgroups of patients with Crohn’s disease divided according to duration of the disease and treatment with biological therapy (BT). Comparison of BJA prevalence (expressed in percentage) between: (**a**) patients with short (up to 1 year), moderate (1–5 years) and long-term (> 5 years) course of the disease; (**b**) patients treated with or without BT upon recruitment to the study; and (**c**) patients with any history of BT and those who have never had any BT. *p*—level of significance.

With regard to gastric abnormalities, no significant differences were observed between the control and study groups with regard to the prevalence of endoscopic features, including ulcerations, polyps or portal gastropathy (Supplementary Table 1). However, we observed that gastric mucosal inflammation and erosions were more common in patients with Crohn’s disease (Table 1 and Fig. 2a). Moreover, BJA of the gastric folds was exclusively observed in patients with Crohn’s disease (Table 1 and depicted on Fig. 2b). Prevalence of this lesion was similar when compared between patients with short (< 1 year), moderate (1–5 years), and long-term (> 5 years) duration of Crohn’s disease (Fig. 3a). There was also no significant difference in BJA prevalence between subgroups of CD patients, who were categorized depending on current treatment with or without biologic agents, as well as, on past history of such treatment (Fig. 3b,c). Notably, among all the initially recruited non-IBD individuals (including those with *H. pylori* infection and those who did not undergo *H. pylori* testing) no patient showed endoscopic lesions that indicated or even resembled BJA. Interestingly, pathology analysis of biopsies taken from BJA lesion demonstrated lack of specific changes and only features indicative of various intensity of chronic gastritis.

### Duodenal lesions

We also evaluated the prevalence of duodenal lesions in this study and observed that duodenal abnormalities were more common in patients with Crohn’s disease than in non-IBD controls**.** Specifically, we observed that mucosal swelling, aphthous lesions, duodenal ulcerations, and bulb deformations were significantly more common in (especially *H. pylori* negative) patients with Crohn’s disease than in controls (Table 1 and Fig. 2c). The prevalence of endoscopic signs indicating presence of intestinal villi flattening, erosions, white deposits, mucosal inflammation and Brunner’s gland hyperplasia did not significantly differ between the control and study groups (Supplementary Table 1). However, statistical analysis showed that with regard to presence of duodenal mucosal inflammation (as depicted on Fig. 2d), values tended to be closer to the level of significance (Supplementary Table 1).

### Microscopic features of chronic gastritis

Collected gastric biopsies were subjected to pathology evaluation. While all of the non-IBD patients with *H. pylori* infection had microscopic features indicative of chronic gastritis, among *H. pylori* negative controls features of gastritis were found in 31% of cases. In comparison, pathology evaluation revealed that almost all—93.5% of the analyzed *H. pylori*-negative patients with Crohn’s disease were found to have features of microscopic chronic gastritis (*p* < 0.00001).

## Discussion

Clinical diagnosis and therapeutic management of Crohn’s disease remain challenging. Conventionally, this disease is known to cause inflammation of the terminal ileum and/or colon. However, studies performed over the last two decades have reported macroscopic abnormalities that tend to commonly affect the more proximal segments of the GI tract, including upper gastrointestinal tract^8^. The exact pathogenesis of such abnormalities remains unknown, and the prevalence of involvement of the upper gastrointestinal tract among patients with Crohn’s disease is also controversial. Therefore, in this prospective study, we focused on evaluation of esophageal, gastric, and duodenal lesions in patients with Crohn’s disease, with particular interest in BJA lesions of the stomach, and compared their prevalence with that observed in non-IBD individuals.

We observed that *H. pylori* infection is significantly less common in patients with Crohn’s disease than in non-IBD adults. While we acknowledge that our findings may be (at least in part) influenced by recruitment bias, as non-IBD individuals included in our study were evaluated due to upper abdominal discomfort and *H. pylori* infection is one of the most common causes of gastritis, still our results are in agreement to previously published data by investigators from other countries^9–11^. This is an interesting finding because the significantly lower prevalence of *H. pylori* infection in patients with Crohn’s disease was earlier attributed to factors other than the disease per se, such as previously administered antibiotic therapy and/or differences in the geographic distribution of this pathogen^12^. However, careful systemic review of available data could not conclusively attribute this phenomenon to the aforementioned potential cofounders^13^. Further studies are necessary to definitively outline the exact mechanisms underlying this trend, as well as, their significance in the development and/or clinical management and outcomes in patients with Crohn’s disease.

Interestingly, in our study, the prevalence of various types of endoscopically documented gastric and duodenal (but not esophageal) mucosal lesions was significantly higher in patients with Crohn’s disease even in the absence of *H. pylori* infection. These lesions primarily included gastric mucosal inflammation, as well as duodenal swelling, ulcerations, and bulb deformation. Our findings are consistent with those reported by previous studies performed in pediatric patients with Crohn’s disease^13,14^, in which it was reported that upper GI involvement may be observed in up to 50% of children with Crohn’s disease, and *H. pylori*-negative gastritis and duodenitis occurred in approximately 26–28% of cases. Furthermore, similar to findings reported by previous studies^5,6^, we observed BJA in the stomach of patients included in our study. This unique abnormality was exclusively observed in patients with Crohn’s disease, in those with and without *H. pylori* infection. In our study, more than 50% of patients showed such lesions in the stomach. Our findings concur with those of previous retrospective studies^7,15^, in which authors observed BJA of the stomach during esophagogastroduodenoscopy in approximately 38–44% of patients with Crohn’s disease. Results of our analyses also reveal that in terms of adults BJA lesion is equally present in patients with short and long-term duration of the disease, and is not associated with applied biological therapy. Moreover, a particularly important highlight of our study is that although all the aforementioned abnormalities were relatively common in patients with Crohn’s disease, despite any clinical symptoms suggestive of upper GI tract involvement and a negative *H. pylori* status, a similar spectrum of endoscopic findings (BJA or duodenal abnormalities) was rare (if at all present) among controls without *H. pylori* infection.

It is valuable to mention that currently little is known about the exact etiology of lesions such as BJA, and factors responsible for particular pattern of their presentation. Pathology analysis of BJA usually reveals presence of hyperplastic fundic glands without metaplasia or atrophy, stromal edema with immune infiltration of the lamina propria toghether with lymphoid follicles and granulomas^5,8^. One may suspect that the BJA presentation pattern of raised and flattened areas may be caused by mucosal expansion caused by ongoing inflammatory process. Notably, what is also a major finding of our study is that nearly all of the patients with Crohn’s disease, and not only a minor portion of them as suggested by others^16^, have microscopic pathologic features indicative of chronic gastric inflammation. Our study demonstrates that this phenomenon is not associated with *H. pylori* infection, and according to available literature *H. pylori*-negative gastritis is generally a rare phenomenon^17,18^. Unfortunately, currently the actual pathomechanisms that contribute to the aforementioned mucosal inflammation in the stomach and throughout the upper GI tract in patients with Crohn’s disease are unknown. However, one cannot exclude the role of an infectious agent that may exclusively or at least partially be associated with such changes occurring on both microscopic and macroscopic levels. This indication is driven by results of our recent molecular study, in which we analyzed the gastric microbiome in *H. pylori—*negative patients with Crohn’s disease. We observed that in comparison to non-IBD control individuals the gut microbiota profile was significantly altered in patients with Crohn’s disease, with differences noted in beta diversity, bacterial phyla, and individual taxa^19^. We consider that the gastric and duodenal mucosal abnormalities reported in this study may represent a consequence/manifestation of the aforementioned altered microbiome. We hypothesize that the endoscopic abnormalities observed in our study may be attributable to some potential factor, such as a specific bacterial agent, a modified bacterial profile, or an altered gastric/duodenal microenvironment secondary to microbiome alterations. Our observations and hypothesis are further supported by results reported by Press and colleagues, who found that in patients with Crohn’s disease the pH profile of the gastric microenvironment is altered in comparison to healthy individuals^20^. However, further translational and clinical research is required to confirm this hypothesis. We are confident that an accurate understanding of this phenomenon will provide better clarity regarding the exact mechanisms underlying the development of mucosal abnormalities of the upper gastrointestinal tract in patients with Crohn’s disease, and also clearly define the pathogenesis of this disease and/or the causative factors.

In summary, our study highlights the low prevalence of *H. pylori* infection in patients with Crohn’s disease. However, despite the absence of *H. pylori* infection, most of these patients show different endoscopic abnormalities involving the upper GI tract, most commonly affecting gastric and duodenal mucosa. Similar endoscopic findings are rarely, if at all, observed in non-IBD *H. pylori* negative adults. BJA of the gastric architecture/folds is exclusively detected in patients with Crohn’s disease independently on *H. pylori* status, duration of the disease and (history of) use of biological therapy. Finally, our study highlights that nearly all of the patients with Crohn’s disease, without *H. pylori* infection have pathologic features of chronic gastritis.

## Material and methods

### Ethics statement/declaration

This study was performed in accordance with appropriate regulations and guidelines highlighted in the “World Medical Association Declaration of Helsinki—Ethical Principles for Medical Research Involving Human Subjects”. The study protocol was approved by the Institutional Bioethical Committee of the Pomeranian Medical University in Szczecin, and all patients provided written informed consent prior to inclusion in the study.

### Study participants and clinical protocols

To this study we prospectively included 375 participants (80 patients with Crohn’s disease and 295 non-IBD individuals) without history of any upper GI surgery or endoscopic intervention. All patients underwent inpatient or outpatient diagnostic upper endoscopic evaluation at our Department. Study participants were categorized into patients with Crohn’s disease (study group) and non-IBD individuals (control group). Patients with Crohn’s disease underwent esophagogastroduodenoscopy as a component of comprehensive evaluation, following initial diagnosis of this condition, for follow-up examination during remission, and/or for evaluation of a flare of the disease. Non-IBD individuals underwent esophagogastroduodenoscopy as a routine diagnostic procedure of dyspeptic symptoms either for initial evaluation, worsening or follow up. Esophagogastroduodenoscopies were performed at our Department by experienced board certified gastroenterologists using high-definition (HD) endoscopy with narrow band imaging (NBI) technology. Close attention was paid to the detection of upper gastrointestinal tract lesions, including BJA. During esophagogastroduodenoscopy biopsy samples were collected from both gastric antrum and fundus/body. One biopsy from antrum was used for the rapid urease test for assessment of *H. pylori* status. Subsequently, biopsy specimens were subjected to evaluation by an experienced board certified pathologist. CD was diagnosed during a standard diagnostic work-up using the Porto criteria, modified in accordance with ECCO guidelines.

As shown in the flow-chart (Fig. 1), among initially recruited individuals in 114 cases no *H. pylori* testing was performed, and a total of 110 patients were found to have *H. pylori* infection. Given that presence of *H. pylori* may contribute towards development of abnormalities in the upper gastrointestinal tract, in this study analyses were performed in two phases—first among all of the recruited 375 individuals irrespectively of *H. pylori* status (80 patients with Crohn’s disease and 295 non-IBD individuals), and subsequently only in patients who tested negative for *H. pylori* (58 patients with Crohn’s disease and 93 non-IBD individuals) The general characteristics of recruited participants has been summarized in Table 2.

**Table 2:** General characteristics of all participants recruited to the study (data presented as means ± SD).

Parameter	All			*H.* *pylori* negative		
	non-IBD individuals (n = 295)	CD patients (n = 80)	*p*	non-IBD individuals (n = 93)	CD patient (n = 58)	*p*
Age (years)	36 ± 10	33 ± 8	0.08	35 ± 9	33 ± 8	0.09
Sex (M-male/F-female)	136-M/159-F	39-M/41-F	0.38	45-M/48-F	28-M/30-F	0.56
(History of) Smoking		9 (11.25%)			8 (13.8%)	
Duration of disease						
Initial diagnosis and/or ≤ 1 year		19 (23.75%)			13 (22.41%)	
1–5 years		24 (30.0%)			18 (31.03%)	
> 5 years		37 (46.25%)			27 (46.55%)	
(History of) Surgery		43 (53.75%)			32 (55.17%)	
Type of disease						
Ileitis		17 (21.25%)			11 (19.0%)	
Ileocolitis		49 (61.25%)			39 (67.24%)	
Colitis		14 (17.5%)			8 (13.8%)	
Disease behavior						
Structuring		49 (61.25%)			38 (65.52%)	
Inflammatory		31 (38.75%)			20 (34.48%)	
CDAI						
Mild		27 (33.75%)			17 (29.3%)	
Moderate		32 (40.0%)			21 (36.2%)	
Severe		21 (26.25%)			20 (34.5%)	
Symptoms (at presentation)						
Abdominal pain		73 (91.25%)			54 (93.1%)	
Changes in bowel habits						
None		18 (22.5%)			12 (20.69%)	
Diarrhea		49 (61.25%)			37 (63.79%)	
Other (constipation, constipation-diarrhea)		13 (16.25%)			9 (15.52%)	
Fever		33 (41.25%)			26 (44.83%)	
Extraintestinal involvement						
Ophthalmic		3 (3.75%)			3 (5.17%)	
Cutaneous		6 (7.5%)			5 (8.62%)	
Articular		18 (22.5%)			17 (29.3%)	
Primary sclerosing cholangitis		2 (2.5%)			2 (3.45%)	
Current use/history of:						
Azathioprine		35 (43.75%)			24 (41.38%)	
Glucocorticoids		43 (53.75%)			31 (53.45%)	
Biologic therapy		33 (41.3%)			22 (37.9%)	

*CD* Crohn’s disease, *CDAI* Crohn’s disease activity index.

### Statistical analysis

All results were subjected to a comprehensive statistical analysis with use of similar protocols as previously^21–25^. Specifically, in case of continuous variables normality of distribution was analyzed with use of the Shapiro–Wilk’s test. Continuous variables that were not normally distributed were subsequently subjected to a logarithmic transformation and their normality of distribution was again evaluated. If normal distribution of the variable was obtained then mean values of examined parameters were compared between the groups with use of the Student’s t-test. Otherwise not normally distributed valuables were compared with use of the Mann–Whitney’s U-test. For comparison of non-continuous variables the Fisher’s exact and Chi-square tests were used. SPSS software was used to conduct all of the aforementioned statistical analysis. Statistical significance was defined as *p* < 0.05.

## Supplementary Information

Supplementary Information.

## Data Availability

The datasets generated during and/or analyzed during the current study are available from the corresponding author on reasonable request.

## References

[1] Feuerstein JD, Cheifetz AS (2017). Crohn’s disease: Epidemiology diagnosis and management. Mayo Clin. Proc..

[2] Cheifetz AS (2013). Management of active Crohn’s disease. JAMA.

[3] Nugent FW, Roy MA (1989). Duodenal Crohn’s disease: An analysis of 89 cases. Am. J. Gastroenterol..

[4] Rutgeerts P (1980). Crohn’s disease of the stomach and duodenum: A clinical study with emphasis on the value of endoscopy and endoscopic biopsies. Endoscopy.

[5] Yokota K (1997). A bamboo joint-like appearance of the gastric body and cardia: Possible association with Crohn’s disease. Gastrointest. Endosc..

[6] Fujiya M (2015). A bamboo joint-like appearance is a characteristic finding in the upper gastrointestinal tract of Crohn’s disease patients: a case-control study. Medicine (Baltimore).

[7] Greuter T (2018). Upper gastrointestinal tract involvement in Crohn’s disease: Frequency, risk factors, and disease course. J. Crohns. Colitis..

[8] Dabkowski K (2019). Clinical significance of endoscopic findings in the upper gastrointestinal tract in Crohn’s disease. Scand. J. Gastroenterol..

[9] Rokkas T, Gisbert JP, Niv Y, O’Morain C (2015). The association between *Helicobacter*
*pylori* infection and inflammatory bowel disease based on meta-analysis. United Eur. Gastroenterol. J..

[10] Wu X-W (2015). Helicobacter pylori infection and inflammatory bowel disease in Asians: A meta-analysis. World J. Gastroenterol..

[11] Shah A (2017). Is there a link between *H.*
*pylori* and epidemiology of Crohn’s disease?. Dig. Dis. Sci..

[12] Amre DK (2006). Investigating the hygiene hypothesis as a risk factor in pediatric onset Crohn’s disease: A case-control study. Am. J. Gastroenterol..

[13] Genta RM, Sonnenberg A (2012). Non-Helicobacter pylori gastritis is common among paediatric patients with inflammatory bowel disease. Aliment. Pharmacol. Ther..

[14] Park JH (2017). Characteristics of upper gastrointestinal tract involvement in Korean pediatric Crohn’s disease: A multicenter study. Pediatr. Gastroenterol. Hepatol. Nutr..

[15] Kuriyama M (2008). Specific gastroduodenoscopic findings in Crohn’s disease: Comparison with findings in patients with ulcerative colitis and gastroesophageal reflux disease. Dig. Liver Dis..

[16] So H (2016). Gastric lesions in patients with Crohn’s disease in Korea: A multicenter study. Intest. Res..

[17] Gantuya B (2019). Gastric microbiota in *Helicobacter*
*pylori*-negative and -positive gastritis among high incidence of gastric cancer Area. Cancers (Basel).

[18] El-Zimaity H, Choi WT, Lauwers GY, Riddell R (2018). The differential diagnosis of *Helicobacter*
*pylori* negative gastritis. Virchows. Arch..

[19] Ostrowski J (2021). The gastric microbiota in patients with Crohn's disease; a preliminary study. Sci. Rep..

[20] Press AG (1998). Gastrointestinal pH profiles in patients with inflammatory bowel disease. Aliment. Pharmacol. Ther..

[21] Deskur A (2014). Selected hemostatic parameters in patients with pancreatic tumors. Am. J. Transl. Res..

[22] Blogowski W (2015). An attempt to evaluate selected aspects of “Bone-Fat Axis” function in healthy individuals and patients with pancreatic cancer. Medicine.

[23] Bodnarczuk T (2018). Hydroxyeicosatetraenoic acids in patients with pancreatic cancer: A preliminary report. Am. J. Cancer Res..

[24] Madej-Michniewicz A (2015). Evaluation of selected interleukins in patients with different gastric neoplasms: A preliminary report. Sci. Rep..

[25] Blogowski W (2016). Interleukins 17 and 23 in patients with gastric neoplasms. Sci. Rep..

