# Supplementary material for: Endoscopic findings in the upper gastrointestinal tract in patients with Crohn’s disease are common, highly specific, and associated with chronic gastritis
Source: Sci Rep. 2023 Jan 13;13:703. doi: 10.1038/s41598-022-21630-5 (PMC9839771; doi:10.1038/s41598-022-21630-5)
Supplement: Supplementary file 1 — Supplementary Information. [file 41598_2022_21630_MOESM1_ESM.pdf]

Supplementary Table 1. Prevalence and types of endoscopic findings among patients with Crohn's disease and healthy individuals together with statistical comparison. Analyses were conducted separately for whole population of recruited individuals and separately for H. pylori negative population of non-IBD and CD patients.

	**All**			***H. pylori* negative**		
	**non-IBD individuals** (n = 295)	**CD patients** (n = 80)	**p**	**non-IBD individuals** (n = 93)	**CD patients** (n = 58)	**p**
Esophagus						
Erosions	6 (2.0%)	0 (0%)	0.23	2 (2.2%)	0 (0%)	0. 83
Ulcerations	2 (0.7%)	0 (0%)	0.62	1 (1.1%)	0 (0%)	0.62
Barrett's metaplasia	6 (2.0%)	0 (0%)	0.23	1 (1.1%)	0 (0%)	0.62
Stomach						
Ulcerations	8 (2.7%)	2 (2.5%)	0.65	1 (1.1%)	2 (3.5%)	0.33
Polyps	8 (2.7%)	3 (3.8%)	0.41	4 (4.3%)	3 (5.2%)	0.55
Portal gastropathy	4 (1.4%)	3 (3.8%)	0.16	1 (1.1%)	2 (3.5%)	0.33
Hyperplasia of gastric folds	30 (10.2%)	11 (4.1%)	0.16	9 (9.7%)	11 (19.0%)	0.08
Duodenum						
Flattening of the intestinal villi	3 (1.0%)	3 (3.8%)	0.11	1 (1.1%)	2 (3.5%)	0.33
Erosions	6 (2.0%)	3 (3.8%)	0.30	1 (1.1%)	3 (5.2%)	0.16

Mucosal inflammation	12 (4.1%)	4 (5.0%)	0.46	0 (0.0%)	3 (5.2%)	0.06
Brunner's gland hyperplasia	0 (0.0%)	1 (1.3%)	0.21	0 (0.0%)	0 (0.0%)	-----

CD – Crohn's disease

IBD – inflammatory bowel disease

p – level of significance
